# RING-PyMOL: residue interaction networks of structural ensembles and molecular dynamics

**DOI:** 10.1093/bioinformatics/btad260

**Published:** 2023-04-20

**Authors:** Alessio Del Conte, Alexander Miguel Monzon, Damiano Clementel, Giorgia F Camagni, Giovanni Minervini, Silvio C E Tosatto, Damiano Piovesan

**Affiliations:** Department of Biomedical Sciences, University of Padua, Padova 35121, Italy; Department of Information Engineering, University of Padua, Padova 35121, Italy; Department of Biomedical Sciences, University of Padua, Padova 35121, Italy; Department of Biomedical Sciences, University of Padua, Padova 35121, Italy; Department of Biomedical Sciences, University of Padua, Padova 35121, Italy; Department of Biomedical Sciences, University of Padua, Padova 35121, Italy; Department of Biomedical Sciences, University of Padua, Padova 35121, Italy

## Abstract

RING-PyMOL is a plugin for PyMOL providing a set of analysis tools for structural ensembles and molecular dynamic simulations. RING-PyMOL combines residue interaction networks, as provided by the RING software, with structural clustering to enhance the analysis and visualization of the conformational complexity. It combines precise calculation of non-covalent interactions with the power of PyMOL to manipulate and visualize protein structures. The plugin identifies and highlights correlating contacts and interaction patterns that can explain structural allostery, active sites, and structural heterogeneity connected with molecular function. It is easy to use and extremely fast, processing and rendering hundreds of models and long trajectories in seconds. RING-PyMOL generates a number of interactive plots and output files for use with external tools. The underlying RING software has been improved extensively. It is 10 times faster, can process mmCIF files and it identifies typed interactions also for nucleic acids.

**Availability and implementation:**

https://github.com/BioComputingUP/ring-pymol

## 1 Introduction

Non-covalent interactions are responsible for the conformational stability and molecular function of biological polymers. The analysis of interaction patterns and their dynamics provides the ground to study the function of proteins (del Sol et al. 2006). Despite their importance, identifying relevant interactions from atomic coordinates is not trivial.

The analytical calculation of structure energy to detect non-covalent interactions is impractical; in particular when analyzing conformations in large structural ensembles or long molecular dynamics (MD). An efficient solution is to detect interactions based on geometrical rules which approximate experimental observation. The residue interaction networks (RINs) generated adopting this approach have been effectively used to analyze protein dynamics and allosteric regulation, for a recent review, see Liang et al. (2020).

An example of software for an efficient calculation of RINs is RING (Martin et al. 2011; Piovesan et al. 2016; Clementel et al. 2022). RING infers contacts and their type (hydrogen bond, ionic bond, π–π stack, etc.) based on a set of simple rules, and it has been recently updated in order to process multi-state structures. A number of other tools can efficiently calculate and analyze protein interactions in multi-state structures (Sladek et al. 2021; Song et al. 2022); however, only few allow to visualize the full set of interactions in a structure viewer and none of them is integrated into PyMOL (Schrödinger, LLC, 2015). Natively, PyMOL is not designed to analyze MD simulations and only a handful of plugins have been designed to process that type of data. For example, PyProGA (Sladek et al. 2021) calculates RINs to identify conformational relationships in dynamic structures exploiting graph mathematics, however, compared to RING, the calculated contacts do not reflect physical principles. Both RING and PyProGA do not show calculated interactions in the structure. PyMOL natively can detect polar (hydrogen bonds) and π–π/π–cation interactions; however, ionic and van Der Waals are missing and the π interactions are imprecise. Moreover, the calculation is performed internally, i.e. it is not accessible via the public API, therefore making it difficult to implement additional calculation without modifying the source code.

Here we present RING-PyMOL, a PyMOL plugin implemented in Python that wraps the RING program (Clementel et al. 2022) and implements additional components to visualize and analyze non-covalent interactions in both single- and multi-state structures. RING-PyMOL combines high-quality non-covalent interactions as provided by RING, with the power of PyMOL to manipulate and visualize protein structures. For multi-state structures, featured RING-PyMOL calculations include RMSD-based clustering and interaction correlations.

The plugin can generate the network either using a local instance of the RING executable or directly querying the RING webserver (Clementel et al. 2022). The RING executable can be freely downloaded from the URL https://biocomputingup.it/download. The RING-PyMOL plugin is available at URL https://github.com/BioComputingUP/ring-pymol.

## 2 Implementation

The RING-PyMOL plugin has a graphical user interface to interact with the PyMOL environment and depends on the RING executable and few standard Python libraries (Pmw, Qt-Material, BioPython, Networkx, Pandas, Seaborn, Scipy, and Numpy).

RING-PyMOL is installed as any other PyMOL plugins by importing the package file or the package folder or just providing the repository link. Interactions are rendered as Compiled Graphics Objects which, since based on OpenGL, is very fast and lightweight, allowing to visualize contacts in structures of any size and number of states seamlessly.

The external RING component has been improved extensively (Clementel et al. 2022). It is now easier to install, it does not require setting environment variables, and the source code has been updated to the C++17 standard. RING now provides typed interactions also for nucleic acid in addition to amino acids and can process mmCIF files natively. The RING algorithm has been optimized and it is about 10 times faster. However, the greatest improvement is that it can now process ensemble conformations (i.e. NMR structure with multiple models) and MD. Multi-state structure analyses are based on the calculation of probabilistic networks and time-dependent network trajectories.

### 2.1 Single-state structures

When working with a single-state structure, e.g. X-ray structure, RING-PyMOL executes RING and immediately shows non-covalent intra- and inter-molecular interactions as lines connecting atoms. The visualization is managed directly at the PyMOL level. In the menu panel, calculated interactions and participating nodes are available as object groups, one for the edges and another for the residues involved in the interaction; different objects correspond to different types of interaction. The plugin allows to modify edge visualization (color, width, and transparency), select intra- or inter-chain interactions and generates a number of helper plots to visualize chain–chain interactions and secondary structure connectivity.

### 2.2 MD simulations and structural ensembles

**Figure 1.**
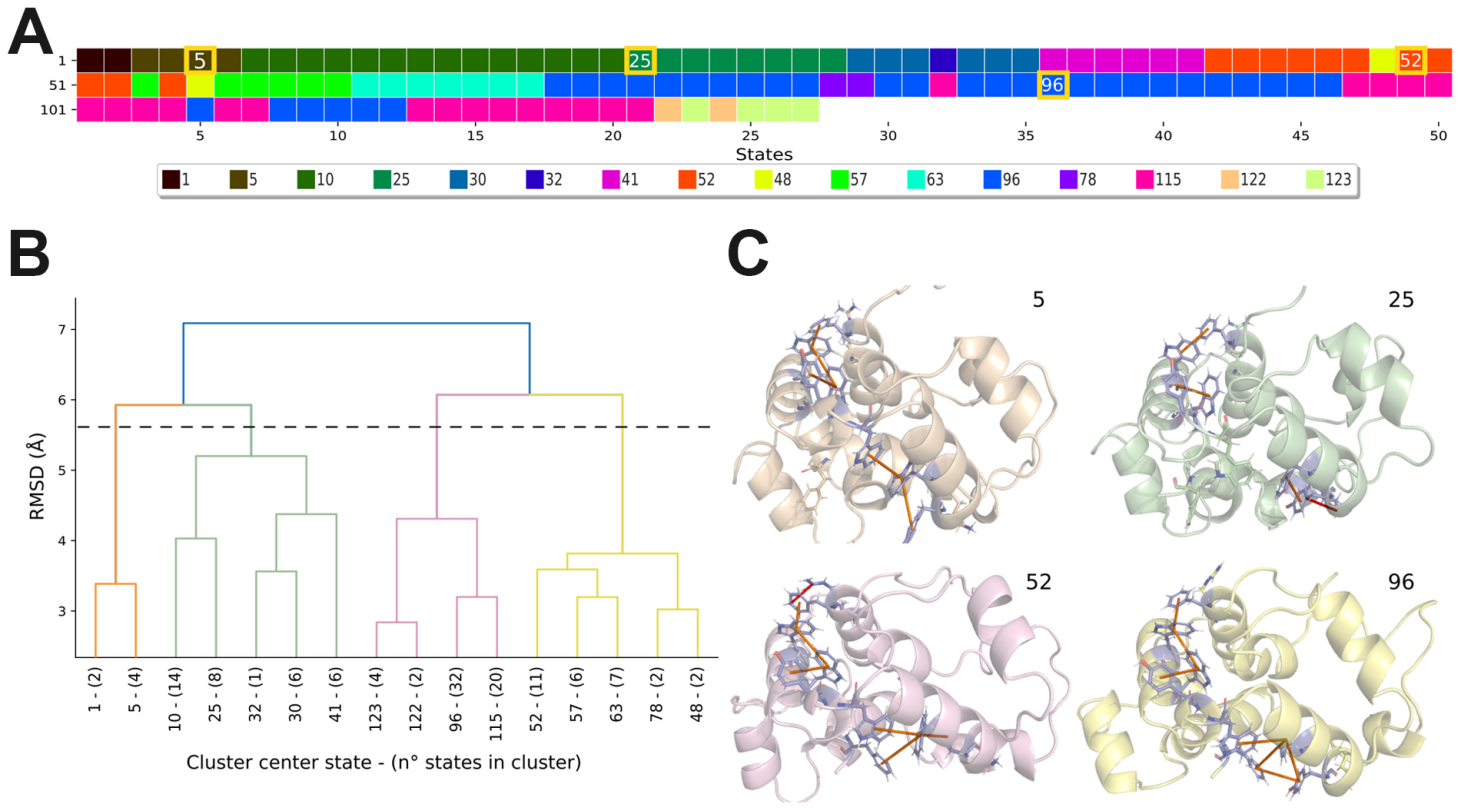
RING-PyMOL analysis of a MD simulation. Snapshots have been derived from a simulation of the STIM1 protein (PDB 2K60) run for 100 ns and sampled every 0.8 ns generating 125 states. (A) Cluster assignments for all states. Different colors represent different clusters. The yellow layout indicates those states shown in (B). The legend indicates the states selected as clusters representatives. (B) RMSD-based structural hierarchy visualized as a dendrogram. The clustering, using default parameters and 2.5 Å cutoff, generated 16 different clusters. The dashed line indicates the cutoff (5.5 Å) used to select the representative conformations shown in (C). (C) Patterns of π–π and π–cation interactions in the selected conformations. Interacting residues are displayed as sticks and colored by element, different states show different contact patterns.

RING-PyMOL offers its greatest power when used with structural ensembles and MD simulations. Multi-state analyses include: (i) visualization of interactions frequencies; (ii) probabilistic contact maps; (iii) contacts correlation statistics; and (iv) structure-based hierarchical clustering. Interaction frequencies are useful to highlight dynamic contacts and to filter out stable contacts, i.e. those not changing over time or across conformations. The probabilistic contact maps are useful to visualize global rearrangements and identify dynamic subregions. Contact correlations (Pearson’s correlation coefficient) are computed on a matrix where the columns represent the states and rows are the number of contacts for each position in the sequence. The statistics can be calculated either considering all interactions or specifically for a single interaction type. Contact correlations are also listed in an interactive table which can be used to focus on subsets of interactions and identify stable (or transient) interactions and pairs of correlating (or anti-correlating) contacts, e.g. those involved in allosteric communication. The hierarchical clustering is useful to reduce the states without losing important information, e.g. when analyzing long MD simulations (Fig. 1). The clustering is calculated on an all-versus-all root-mean-square deviation (RMSD) matrix obtained after superimposing all the conformations with the Bio.PDB.Superimposer module of BioPython. The structure hierarchy is provided as a dendrogram while cluster assignments are plotted in a heatmap to visualize conformational transitions along the simulation. Representative conformations are stored in standard PyMOL structure objects and can be used as a starting point for further analyses.

## 3 Summary

We implemented RING-PyMOL to easily visualize non-covalent interaction patterns in multi-state structures and molecular dynamic trajectories directly in the PyMOL viewer. Contact correlations, structural clustering in relation to time can highlight significant conformational rearrangements, and identify key residues, functional sites, and allosteric communication pathways.
